# Dimensional Accuracy Assessment of 3D-Printed Edentulous Jaw Models: A Comparative Analysis Using Three Laboratory Scanners

**DOI:** 10.3390/ma18143323

**Published:** 2025-07-15

**Authors:** Spartak Yanakiev, Mariana Dimova-Gabrovska

**Affiliations:** 1 Medical College “Y. Filaretova”, Medical University—Sofia, Yordanka Filaretova 3 Str., 1606 Sofia, Bulgaria; 2 Department of Prosthetic Dentistry, Faculty of Dental Medicine, Medical University—Sofia, Georgi Sofiiski Str., 1431 Sofia, Bulgaria; prof.marianadimova@gmail.com

**Keywords:** laboratory scanner, dimensional accuracy, additive manufacturing, trueness, precision, edentulous jaw models

## Abstract

The dimensional accuracy of 3D-printed edentulous jaw models is critical for successful prosthetic treatment outcomes. This study investigated the accuracy of 3D-printed working models of a completely edentulous jaw through comparative analysis of digital images generated by three laboratory scanners. A reference plaster model of a mandibular edentulous arch was digitized and used to produce ten resin models via digital light processing (DLP) technology. Each model was scanned using three different laboratory scanners: AutoScan-DS-EX, AutoScan-DS-EX Pro(H), and Optical 3D Scanner Vinyl. Digital comparison was performed using specialized software, evaluating the root mean square (RMS) deviation and percentage of values within an acceptable deviation range ±0.05 mm. All printed models showed significant deviations from the reference model (*p* < 0.05), with RMS values ranging from 109.2–139.7 µm and acceptable deviation percentages ranging from 29.34 to 32.31%. The mean precision RMS value was 66.37 µm. The mean intraclass correlation coefficient of 0.544 indicated moderate precision. Optical 3D Scanner Vinyl demonstrated the highest consistency, while AutoScan-DS-EX Pro(H) showed maximum variability. No statistically significant differences were found between scanners (*p* = 0.075). While the investigated scanners demonstrated reliable performance and sufficient accuracy, the additive manufacturing process introduced clinically significant deviations, highlighting the importance of verification between printed models and their digital originals before proceeding with clinical stages. Clinical practice should prioritize scanning systems with advanced software algorithms over those with superior hardware specifications alone.

## 1. Introduction

### 1.1. Background

The accuracy of working models is essential for successful prosthetic treatment. Models can be fabricated in physical (plaster/polymer) or digital formats from either digital or conventional impressions. Despite the progressive integration of digital workflows in modern prosthodontics, physical models remain indispensable, particularly for complete denture fabrication.

Model accuracy is determined by two key parameters: trueness and precision. These parameters are relevant to both scanning processes and 3D printing technologies. Trueness measures the degree of correspondence between the final product (digital image or printed model) and the original, quantifying deviation from the physical object. Precision represents reproducibility across multiple iterations, whether in repeated scans or serial model production.

In digital dental medicine, working model accuracy assessment typically focuses on root mean square (RMS) values. The RMS deviation measures the average difference between a set of values and a reference value. It is calculated by squaring the differences, averaging them, and then taking the square root. In 3D scanning or prosthetics, it quantifies how closely a scanned model matches the reference model, with a lower RMS indicating a better fit. Comparative analysis software can also generate a more clinically relevant metric—the percentage of values within the acceptable deviation range—which has received limited attention in the research literature. This percentage metric offers additional clinical utility by directly indicating what proportion of the model surface falls within predetermined clinically acceptable thresholds, enabling practitioners to make immediate assessments of model suitability for specific prosthetic applications.

The accuracy of plaster and printed working models is a key issue for high-quality prosthetic construction. Several studies have demonstrated the advantages of printed models. Nulty et al. [1] confirmed minimal accuracy differences between plaster and printed models, with printed models offering superior results in certain applications due to enhanced stability and resistance to moisture and deformation. Czajkowska et al. [2] reported no statistically significant accuracy differences between intraoral scans and plaster models. Furthermore, printed models based on intraoral scans exhibit superior mechanical properties and greater accuracy compared to traditional plaster models. These findings underscore the advantages of 3D printing in dental model production, particularly regarding durability and detail resolution.

Although printed models offer well-documented benefits, their production process is intricate and requires meticulous management of various parameters. While automation has significantly simplified the practical aspects of model fabrication, several factors impact accuracy. These include the type of 3D technology and printer used [3], mesh resolution [4], model orientation on the platform [5], storage conditions and methods [6], resin type and composition [7], model wall thickness, presence of support structures [8,9,10], and post-processing techniques [7,11,12,13]. Kim J.H. et al. [13] demonstrated that post-polymerization conditions significantly affect physical properties and the dimensional accuracy of 3D-printed dental materials. Furthermore, Väyrynen et al. [14] reported anisotropic flexural properties in stereolithography-processed materials, which could influence the final model quality. Deviations resulting from these factors can accumulate, potentially leading to significant inaccuracies in the working model.

Functional impression-taking for complete dentures remains an aspect of prosthodontics that cannot be entirely digitalized as it requires functional testing. However, the workflow can be digitized by scanning either the functional impression or its corresponding plaster model. The scanner’s role is crucial in this digitization process. Research by Kang et al. [15] revealed significant variations in scanning accuracy depending on scanner type, with higher accuracy observed in posterior regions compared to anterior areas. Borbola D et al. [16] also reported statistically significant differences in precision and trueness among the investigated scanners, though all examined laboratory scanners met clinical usage requirements.

The scientific literature often explores the accuracy of intraoral scanners, while studies on laboratory scanners are relatively fewer [17]. Most studies focus on complete dentate arches, while the printing and digitization of edentulous jaw models remains understudied [18,19,20]. Zarone et al. [21] performed a comparative analysis between chairside intraoral scanners and laboratory scanners for edentulous maxilla, highlighting specific challenges in digitizing edentulous areas. The absence of distinct anatomical landmarks and the presence of smooth, uniform surfaces in edentulous ridges create particular difficulties for optical scanning systems. Lai et al. [22] identified critical considerations regarding additive manufacturing technologies’ effects on trueness and the dimensional stability of printed dies relevant to edentulous model production.

Complete edentulism affects substantial global populations, with prevalence rates of 3–7% in developed countries and exceeding 20% in certain regions, particularly among elderly populations [23]. Given this widespread prevalence, the limited research on the scanning and printing accuracy of edentulous models represents a critical knowledge deficit in digital prosthodontics. Accurate transfer of edentulous jaw morphology is essential for complete denture success, as minor dimensional deviations significantly impact prosthesis fit, stability, and patient comfort, necessitating focused investigation in this specific area.

The limited available studies analyzing the accuracy of laboratory scanners in digitizing working models of completely edentulous jaws and the accuracy of printed working models of edentulous jaws provide justification for further investigations.

### 1.2. Objective

This study aims to evaluate the accuracy of 3D-printed working models of a completely edentulous jaw through a comparative analysis of digital images generated by three laboratory scanners. The null hypotheses are as follows: (1) the 3D-printed models are dimensionally identical to the original plaster reference model and exhibit no significant differences among themselves; (2) there are no statistically significant differences in dimensional accuracy among the digital images generated by the three laboratory scanners.

## 2. Materials and Methods

### 2.1. Study Design and Reference Model Preparation

**Figure 1:**
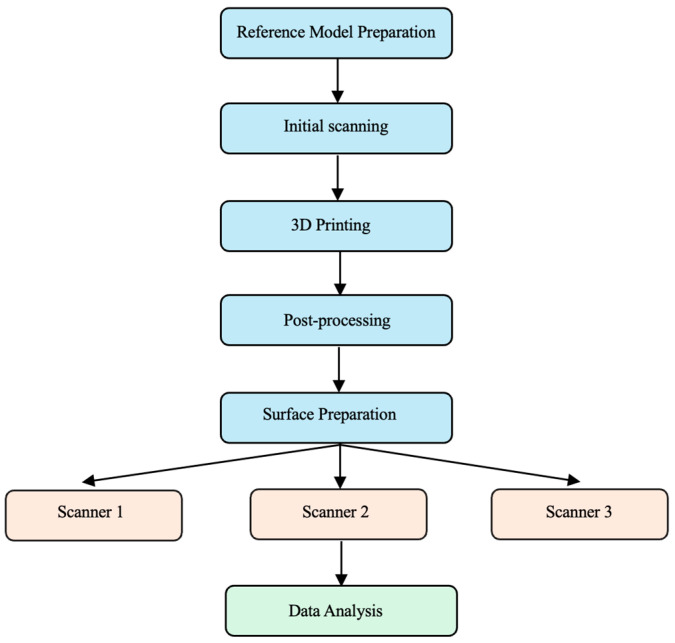
Study design for evaluating the accuracy of 3D-printed edentulous jaw models.

An in vitro comparative analysis of dimensional accuracy in 3D-printed edentulous jaw models was conducted using three laboratory scanners. (Figure 1).

A reference edentulous jaw model was fabricated using Type IV dental stone (Elite Rock, Zhermack SpA, Badia Polesine, Italy) under controlled temperature (23 ± 1 °C) conditions according to the manufacturer’s recommendations for water/powder ratio. Three reference points were strategically placed along the alveolar ridge—one at the midline and two in the molar regions. These 2 × 2 × 4 mm wax parallelepipeds were designed to facilitate precise alignment and measurement during digital comparison. Preliminary testing confirmed their optimal positioning, ensuring reliable scanner recognition with minimal interference with surface geometry.

### 2.2. Digital Workflow and Model Production

The reference model was digitized using the AutoScan-DS-EX Pro(H) scanner (Shining 3D Tech Co., Ltd., Hangzhou, China) following a standardized protocol under controlled lighting conditions to minimize artifacts. Test models (n = 10) were 3D printed using a DLP printer with Anycubic Standard Resin V2 Gray (405 nm) (Shenzhen, China), following the manufacturer’s specifications with a wall thickness of 2 mm. The printing parameters used are presented in Table 1. All models were printed at a 45° orientation to the build platform to optimize detail reproduction and minimize support requirements. The models were sequentially numbered from 1 to 10.

### 2.3. Model Preparation and Processing

The printed models were cleaned in an ultrasonic bath with 95% isopropyl alcohol for 15 min, then dried and subjected to additional polymerization in a combined cleaning and polymerization device, MERCURY PLUS 2.0 (ELEGOO Inc., Beijing, China), for 5 min. The post-curing and cleaning procedures were performed by the same operator with over three years of practice. Before scanning, AESUB white scanning spray (Scanningspray Vertriebs GmbH, Recklinghausen, Germany) was applied to the models, providing approximately 7 µm coverage for two of the scanners used in the study.

### 2.4. Scanning Equipment

The prepared models were scanned, and the generated STL files were saved for subsequent processing. The reference plaster model was also scanned with each of the three scanners. Powder coating was applied only to the wax element areas in order to reduce the reflections of the material. All scans were performed under identical conditions. Table 2 presents the technical specifications of the three scanners used in the study and their specific preparation requirements.

### 2.5. Digital Analysis Methodology

Digital model comparison was performed using specialized software—Medit Link v. 3.3.2 and Medit Design v. 2.1.4 (Medit Corp., Seoul, Republic of Korea). The digital file of the plaster model served as the reference, against which the scans of the ten printed models were compared using the “Automatic alignment” function. Two key parameters were assessed:

1. Root mean square (RMS) deviation in millimeters, subsequently converted to micrometers;
2. Percentage of values within the acceptable deviation range (±0.05 mm, green color).

**Figure 2:**
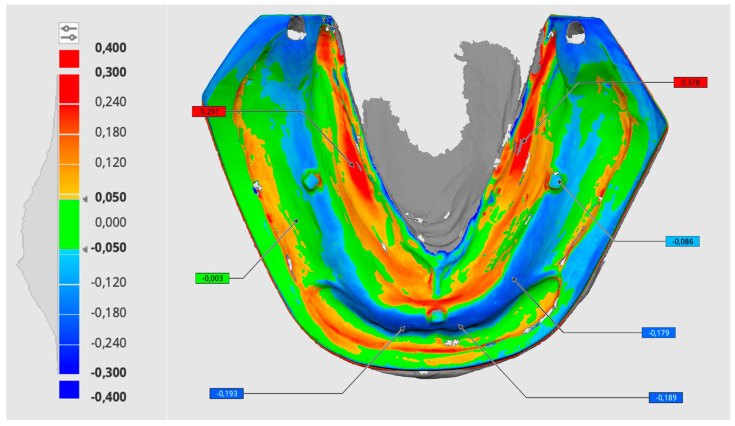
Color map of model accuracy evaluation in Medit Design v. 2.1.4 software. Green areas represent values within the acceptable deviation range. Positive deviations (larger than the reference) are marked in red, and negative deviations (smaller than the reference) are marked in blue.

Maximum deviations were set at ±0.4 mm, with positive deviations (larger than the reference) marked in red and negative deviations (smaller than the reference) marked in blue (Figure 2). For each test/reference pair, ten automatic superimpositions were performed, resulting in a total of 600 measurements (200 per scanner, 100 per parameter) for statistical analysis. Additionally, the scans of the printed models obtained by each of the three scanners were superimposed to evaluate the precision of the models.

### 2.6. Statistical Analysis

Data analysis was performed using IBM SPSS Statistics 27.0.1.0 (IBM Corporation, Armonk, NY, USA) with the significance level set at *p* < 0.05. The following statistical methods were applied: analysis of variance (ANOVA), the Shapiro–Wilk test to verify the normality of the data distribution, the Friedman test for multiple dependent samples comparison to assess differences across the three scanners, and the Wilcoxon signed-rank test for pairwise comparisons between groups.

The precision of the printed models was assessed using Intraclass Correlation Coefficient (2.1) (ICC) analysis to quantify overall model group consistency and correlation analysis was conducted to examine the relationship between RMS and acceptable deviation percentage metrics across all three scanners.

## 3. Results

### 3.1. Model Trueness

The statistical analysis revealed significant differences between the reference plaster model and all printed specimens across all three scanners (*p* < 0.05). The observed deviations (109.2–139.7 µm) vary depending on the model number and the scanner. Results are presented in Table 3, Table 4 and Table 5.

Across all models, Model 1 achieved the lowest RMS value (117.1 µm), while Model 8 showed the highest (137.6 µm). Model 4 demonstrated the most consistent performance with the lowest standard deviation (0.493 µm) based on RMS values across all three scanners, while Model 5 showed the highest standard deviation (8.603 µm), indicating the greatest variations in RMS values between scanners. The highest percentage of acceptable deviation matches across all three scanners was observed in Model 1, with Model 8 showing the lowest match. Results are shown in Table 6.

### 3.2. Model Precision

#### 3.2.1. RMS (Root Mean Square)

The RMS parameter analysis revealed distinct patterns across the three scanners. The highest number of statistically identical mean values was observed in AutoScan-DS-EX, followed by Optical 3D Scanner Vinyl, while AutoScan-DS-EX Pro(H) showed significant differences in mean values between all specimens (Table 3).

#### 3.2.2. Acceptable Deviation

Statistically significant differences between all specimens were observed again in AutoScan-DS-EX Pro(H) (statistical differences between 9 model groups) while Scanners 1 and 3 identified fewer distinct groups (Table 3).

#### 3.2.3. ICC, Confidence Interval (CI), and Correlation Analysis of RMS and Acceptable Deviation Percentage Metrics

The mean ICC (0.544) across the three scanners demonstrated moderate overall precision of the 3D-printed models. The average RMS deviation between models (65.4 μm) remains within the clinically acceptable thresholds for dental prosthetic applications.

##### AutoScan-DS-EX Data Analysis

The analysis conducted with AutoScan-DS-EX yielded the lowest mean RMS values among all scanners (60.6 ± 13.7 μm, range: 32.0–82.0 μm) with moderate inter-model consistency (ICC = 0.539, 95% CI: 0.411–0.689). The acceptable deviation percentage measurements (mean approximately 65%) confirmed exceptional precision between model pairs 1–6 (89.7%), 6–7 (91.9%), and 4–8 (90.6%), demonstrating a strong negative correlation with RMS values (r = −0.92, *p* < 0.001).

##### AutoScan-DS-EX Pro(H) Data Analysis

The precision evaluation using AutoScan-DS-EX Pro(H) revealed moderate consistency (ICC = 0.642, 95% CI: 0.514–0.776) between the ten printed models, with a mean RMS deviation of 73.3 ± 11.3 μm (range: 53.0–92.0 μm). The acceptable deviation percentage metric corroborated these findings, showing a mean value of 62.8 ± 11.3% (range: 43.5–86.1%) with the highest correspondence between specific model pairs (1–6: 86.1%, 1–7: 83.6%), demonstrating a significant negative correlation with RMS values (r = −0.93, *p* < 0.001).

##### Optical 3D Scanner Vinyl Data Analysis

Optical 3D Scanner Vinyl measurements indicated comparatively lower mean RMS values (62.2 ± 16.0 μm, range: 30.0–86.0 μm) but with increased variability and poor inter-model consistency (ICC = 0.451, 95% CI: 0.319–0.621). The acceptable deviation percentage analysis substantiated these observations, maintaining the pattern of highest precision between the same model pairs identified by RMS analysis (1–6: 91.7%, 1–7: 90.5%, 4–8: 92.8%).

### 3.3. Scanner Trueness Comparison

The differences between the three scanners reflect the influence of scanning technology and methodology on result accuracy. The results (mean values and standard deviations of the two examined parameters) are presented in Table 7.

AutoScan-DS-EX Pro(H) demonstrated the lowest mean RMS value (118.97 µm) across all models, indicating that its generated images most closely matched the original model. Scanners 1 and 3 showed very similar mean RMS values (125.51 µm and 125.03 µm, respectively), suggesting comparable performance but with greater deviation from AutoScan-DS-EX Pro(H)’s results.

AutoScan-DS-EX data showed the highest mean acceptable deviation value (32.21%), indicating that this scanner generated digital models with the best correspondence to the original. Optical 3D Scanner Vinyl showed similar but slightly lower values (31.66%), while AutoScan-DS-EX Pro(H) demonstrated the lowest mean acceptable deviation value (29.35%). This suggests that despite its lower RMS values, AutoScan-DS-EX Pro(H) may achieve less successful overall model adaptation.

Analysis of standard deviations based on mean values across the ten models revealed that Optical 3D Scanner Vinyl demonstrated the highest consistency, exhibiting the lowest standard deviation in both RMS (SD = 5.8 µm) and acceptable deviation percentage (SD = 1.96%). Conversely, AutoScan-DS-EX Pro(H) showed the highest variability, with the largest standard deviations in both RMS (7.8 µm) and acceptable deviation percentage (3.15%), indicating greater measurement inconsistency between different models. The observed differences between scanners yielded a *p*-value of 0.075, indicating no statistically significant difference at the predetermined significance level (*p* < 0.05) (Table 7).

**Figure 3:**
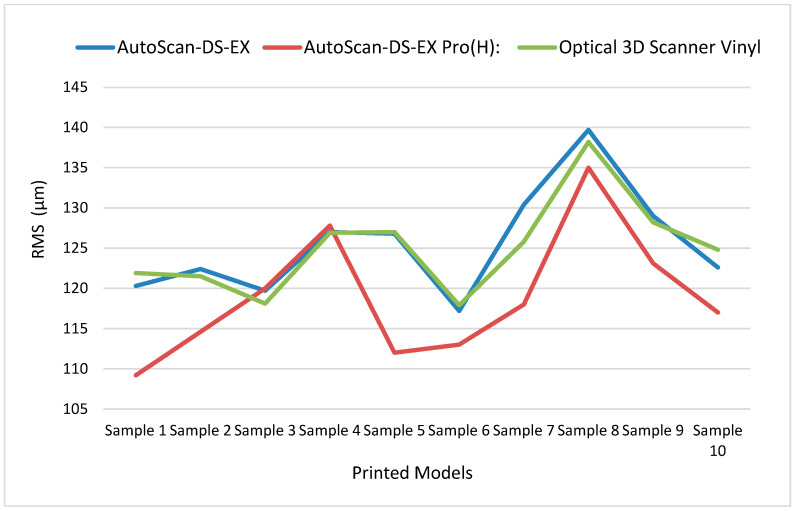
Scanner trueness comparison based on RMS values in µm.

**Figure 4:**
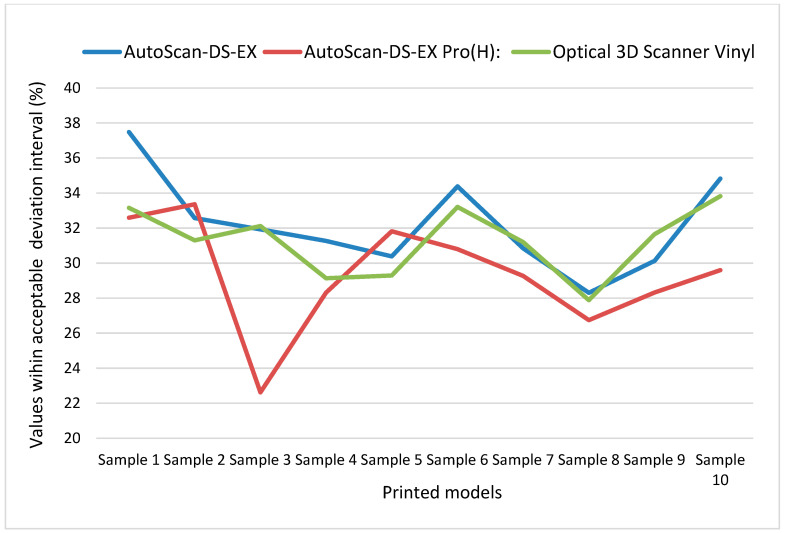
Comparison of scanner trueness based on values within the acceptable deviation interval in percentages.

The graphical comparative variability of the results based on the evaluated parameters is presented in Figure 3 and Figure 4.

## 4. Discussion

This study investigated the accuracy of 3D-printed working models of a completely edentulous jaw through the comparative analysis of digital images generated by three laboratory scanners. The findings reveal complex interactions between scanning technology, printing processes, and accuracy assessment methodologies.

The evaluation of accuracy (trueness and precision) is most often conducted through digital comparison using specialized software or by assessing linear measurements [24,25,26]. RMS values used as assessment parameters in this study align with established objective criteria in digital dentistry research [24,25]. An acceptable deviation percentage was evaluated for the first time in this investigation. Most previous studies rely exclusively on RMS data analysis, which quantifies average deviation magnitude but provides no information on deviation distribution across model surfaces. Our dual-metric approach offers complementary perspectives: RMS values measure absolute deviation magnitude, while acceptable deviation percentages reveal the proportion of the model surface falling within clinically tolerable thresholds (±0.05 mm). This methodology provides clinically relevant information by distinguishing between models with similar RMS values but different deviation distributions—a critical distinction for assessing functional suitability. The comparative analysis of three scanners using both metrics reveals how scanning technology influences not only overall accuracy (RMS) but also clinical acceptability (the percentage of the surface within tolerance), offering more comprehensive guidance for practical implementation in prosthodontic workflows. The color mapping visualization offered by many software packages further enables precise identification of specific regions where the model significantly deviates from acceptable limits. This allows clinicians to focus attention on critical areas requiring adjustment during prosthesis fabrication and make targeted corrections to improve fit accuracy.

### 4.1. Model Trueness

Comparing the trueness of printed models against the original plaster model revealed statistically significant deviations in all ten models across all scanners. Trueness deviations varied by scanner from 109.2 µm to 139.7 µm. Mean trueness values for the printed models were 125.5 ± 6.5 µm, 118.9 ± 7.8 µm, and 125.0 ± 5.8 µm for scanners 1, 2, and 3, respectively. The largest absolute difference was observed in Model 1, with a 12 µm variation between AutoScan-DS-EX Pro(H)’s and Optical 3D Scanner Vinyl’s digital files. However, comparing the data generated by the three scanners revealed statistically insignificant differences that would not be of clinical importance. These findings support the hypothesis that deviations are primarily due to model printing rather than scanning preparation or scanner type. The results emphasize the importance of factors reported in the scientific literature such as optimizing printing parameters, appropriate equipment selection, model design, and printing material choice [3,4,5,6,7,8,9,10,11,12,13].

The scientific literature demonstrates considerable variation in printer accuracy, with reported values ranging from 3.3 μm to 579 μm for SLA printers and approximately 100 μm for DLP printers [1]. Our findings fall within this range and align with previous studies of DLP-printed models, such as those reported by Kim SY et al. (99.8 μm ± 47.2 μm) [9] and Yousef H et al. (153.7 ± 25.4 μm) [6]. Morón-Conejo et al. [26] reported significantly higher RMS values exceeding 245 µm when evaluating different 3D printing workflows for dental models comparing industrial and desktop printers.

The deviations observed in this study (109.2–139.7 µm) demonstrate both the capabilities and limitations of current digital dental technologies. These values exceed the 100 µm threshold generally accepted in prosthetic dentistry, suggesting clinical significance [27]. The values are within the acceptable deviations according to other studies in the field of removable prosthodontics of 300 µm [28]. Al-Dulaijan YA et al. consider accuracy deviations within the range of 50–200 µm to be clinically acceptable but emphasize that in critical areas, such as the intaglio surface or borders, the threshold is 100 µm [29]. However, considering that the natural resiliency of mucosa significantly exceeds that of natural teeth, deviations near the 100 µm threshold need additional investigation, including clinical evaluation of final prosthetic accuracy, which lies beyond the scope of this study. Variations in bone structure and mucosal thickness must be considered [18]; thin mucosa may render these deviations clinically significant, while thicker tissues may compensate for such discrepancies.

### 4.2. Model Precision

Precision assessment in the scientific literature demonstrates deviations ranging from 23–99 μm across different printing technologies [3]. This investigation yielded mean values of 60.6 ± 13.7 μm (AutoScan-DS-EX), 73.3 ± 11.3 μm (AutoScan-DS-EX Pro(H)), and 62.2 ± 16.0 μm (Optical 3D Scanner Vinyl). These measurements exceed those reported by Emir and Ayyildiz [30], who documented mean deviations of 30.4 μm for PolyJet technology, compared to 37.6 μm for SLA and 43.6 μm for DLP technologies. Conversely, our findings are substantially lower than the deviations documented by Morón-Conejo et al. [26], who reported values exceeding 244.81 μm for DLP printing systems. Similarly, our measurements are significantly lower than the 120 ± 20 μm reported by Tsolakis et al. [31] for DLP technologies.

The mean ICC across the three scanners (0.544, range: 0.451–0.642) demonstrates moderate precision according to established interpretation criteria. Statistical guidelines define ICC values below 0.50 as poor reliability, 0.50–0.75 as moderate reliability, 0.75–0.90 as good reliability, and above 0.90 as excellent reliability [32]. An ICC value of 0.544 falls within the moderate reliability range, indicating acceptable but suboptimal consistency among the 3D-printed models. These values are lower than those reported by Rungrojwittayakul et al. [27], who documented superior ICC values exceeding 0.968 for DLP printers regardless of model configuration (hollow-base or solid-base designs).

Strong negative correlations between RMS and acceptable deviation percentage metrics were observed across all scanners (r = −0.92 to −0.93, *p* < 0.001), validating the complementary nature of these assessment approaches.

From a clinical perspective, the measured precision values (60.6–73.3 μm) fall within acceptable limits for dental prosthetic applications, remaining substantially below the critical 100 μm threshold for clinical significance. However, the moderate precision (ICC = 0.544) indicates considerable inter-model variability, requiring quality verification of individual printed models before clinical use. Natural mucosal resilience may partially compensate for these precision deviations in edentulous patients, especially those with thicker tissue profiles.

### 4.3. Scanner Trueness

The assessment of scanner accuracy has been investigated by other research groups [15,16,24]. Statistically significant differences between scanners have been reported, with deviations exceeding 100 μm considered threshold values from a clinical perspective, determining scanner suitability [15]. The type of prosthetic construction and working model is significant, as accuracy deviations increase for complete dental arch applications [33].

Our study data were obtained by comparing digital models of identical physical specimens using three different scanners, which to the best of our knowledge, have not been studied before: AutoScan-DS-EX, AutoScan-DS-EX Pro(H), and Optical 3D Scanner Vinyl. The number of models is in accordance with other studies [1,5,21] and ensures the minimum specimen number for reliable statistical analysis.

The evaluation of scanner precision typically employs superimposition of files obtained from tested and reference scanners. This study utilized an alternative approach by comparing values of identical objects generated by different scanners, which does not provide information about absolute deviation values against a defined accuracy standard but provides comparative accuracy data between devices. The reference STL file was generated from the physical plaster model, while ten test STL files for each scanner were generated from identical printed models. Because all printed specimens originated from the same digital STL data, observed differences primarily reflect printing process deviations rather than design variations. STL file generation for both plaster and printed models was influenced by scanner specifications (software algorithms, hardware parameters, settings), specimen preparation protocols, and comparison software characteristics. These individual factors were beyond the scope of this investigation.

This study employed three different scanners with two model preparation protocols: with and without powder coating. Scanners 1 and 3 generated comparable measurements despite variations in resolution, light source, and preparation methodology. The similarity in results suggests these factors had a minimal impact on scanning outcomes. Both scanners utilize single-camera configurations, which, combined with software algorithms and data processing protocols, likely contributed to the observed consistency. AutoScan-DS-EX achieved comparable performance to Optical 3D Scanner Vinyl despite its inferior technical specifications.

AutoScan-DS-EX’s superior matching percentage indicates optimized data processing algorithms that compensate for digitization artifacts. This finding is consistent with Son et al. [25], who demonstrated significant software influence on scanner accuracy independent of hardware specifications. Optical 3D Scanner Vinyl’s minimal variability establishes it as a reliable device, attributed to hardware characteristics and software integration. AutoScan-DS-EX Pro(H), despite its superior hardware specifications (dual 5-megapixel cameras), exhibited reduced matching percentages, demonstrating the complex relationship between camera resolution and measurement accuracy. This complexity aligns with Galeva et al. [24], who established that optical scanner accuracy depends on multiple interrelated factors beyond hardware specifications alone.

The present study has certain limitations that should be considered. The use of a single printing material and printer type limits the generalizability of the findings. Environmental factors and operator experience, while controlled, may influence real-world applications. One of the limitations of the best fit algorithms is that they tend to underestimate the real discrepancies found when searching for the maximum fit between the surfaces. Factors related to scanner hardware characteristics and associated software also influenced the obtained data. Future research should investigate the impact of different materials, printing technologies, printer settings, and post-processing protocols on model accuracy.

Based on the obtained results, the first null hypothesis was rejected, demonstrating significant deviations between the printed specimens and the reference model with respect to both precision and trueness parameters. The second null hypothesis was accepted, as no statistically significant differences were identified among the digital files generated by the three scanning systems.

### 4.4. Clinical Implications

Based on the study results, the following clinical applications can be derived.

Scanner selection should prioritize consistency over technical specifications alone, with a literature review of specific scanner performance studies recommended when available. AutoScan-DS-EX achieved comparable results to Optical 3D Scanner Vinyl despite inferior specifications, while AutoScan-DS-EX Pro(H)’s advanced hardware yielded suboptimal outcomes. Scanners with optimized software algorithms are preferable to those relying solely on superior hardware specifications.

Working model printing may introduce clinically significant deformations affecting both trueness and precision. To control accuracy, comparison between the digital STL file and the STL file of the printed model is recommended at the initial stage of prosthetic work. This verification protocol enables early detection of dimensional discrepancies before clinical implementation.

Given that the printing process, rather than digitization or scanner type, primarily determines accuracy, practitioners should focus on strict control of 3D printing parameters. Critical factors include printing method, software and hardware characteristics, printer settings, resin selection, and adherence to manufacturer instructions, model orientation, wall thickness, etc. These parameters, which are extensively discussed in the scientific literature, require systematic optimization for consistent clinical outcomes.

Although the observed accuracy deviations are statistically significant, they may remain clinically acceptable for many edentulous applications. The moderate precision values (ICC = 0.544) suggest acceptable reproducibility for routine prosthetic procedures, while natural mucosal resilience may compensate for minor dimensional variations in edentulous patients.

## 5. Conclusions

The present investigation revealed statistically significant deviations (*p* < 0.05) between the reference plaster model and all 3D-printed specimens across the three evaluated scanners, with root mean square (RMS) values ranging from 109.2 to 139.7 µm. Among the tested specimens, Model 1 exhibited superior trueness with a mean RMS value of 117.1 µm, whereas Model 8 demonstrated the highest deviation at 137.6 µm.

The precision analysis yielded a mean intraclass correlation coefficient (ICC) of 0.544, indicative of moderate inter-model reproducibility. Comparative evaluation of scanner performance revealed that AutoScan-DS-EX exhibited the lowest mean RMS values (60.6 ± 13.7 µm), followed by Optical 3D Scanner Vinyl (62.2 ± 16.0 µm) and AutoScan-DS-EX Pro(H) (73.3 ± 11.3 µm). Correlation analysis demonstrated a strong inverse relationship between RMS values and acceptable deviation percentages across all scanners (r = −0.92 to −0.93, *p* < 0.001).

Statistical analysis of inter-scanner variability yielded no significant differences among the three scanning systems (*p* = 0.075). Notably, AutoScan-DS-EX Pro(H) exhibited the lowest mean RMS values (118.97 µm) yet paradoxically demonstrated the lowest acceptable deviation percentage (29.35%). Optical 3D Scanner Vinyl displayed superior measurement consistency, evidenced by minimal standard deviations for both RMS (5.8 µm) and acceptable deviation percentage (1.96%) parameters. The overall mean RMS deviation of 65.4 µm between models remains well within the established clinical tolerance thresholds for prosthetic dentistry applications.

This study presents the first comparative analysis of AutoScan-DS-EX, AutoScan-DS-EX Pro(H), and Optical 3D Scanner Vinyl specifically for edentulous jaw applications, introducing a novel dual-metric assessment approach that combines traditional RMS measurements with acceptable deviation percentage analysis to provide more comprehensive clinical evaluation than conventional RMS-only methods. The research addresses a significant gap in the literature regarding scanning accuracy for edentulous models, as most existing studies focus on dentate arches, while demonstrating that optimized software algorithms can effectively compensate for inferior hardware specifications, evidenced by AutoScan-DS-EX achieving results comparable to technically superior scanners.

Clinical practice should prioritize scanning systems with advanced software algorithms over those with superior hardware specifications alone. Working model printing may introduce clinically significant deformations affecting both trueness and precision. To control accuracy, comparison between the digital STL file and the STL file of the printed model is recommended at the initial stage of prosthetic work. The additive manufacturing process demonstrates a greater impact on dimensional accuracy than scanner selection, though the observed deviations may be clinically acceptable for edentulous prosthetic applications due to the compensatory effect of mucosal tissue resilience.

## Figures and Tables

**Table 1:** Printing parameters of the models.

Parameter	Value
Layers Thickness (mm)	0.05
Normal Exposure Time (s)	3.00
Off Time (s)	1.00
Bottom Exposure Time (s)	50.00
Bottom Layers	5
Anti-alias	16
Gray Level	0
Image Blur	0
Control Type	Basic
Z Lift Distance (mm)	6.00
Z Lift Speed (mm/s)	4.00
Z Retract Speed (mm/s)	6.00

**Table 2:** Technical characteristics of the scanners used in the study.

Scanner	Manufacturer	Cameras	Resolution	Light Source	Powder Coating
Scanner 1—AutoScan-DS-EX	Shining 3D Tech Co., Ltd.	1 camera × 1.3 megapixels	15 µm	White light	No
Scanner 2—AutoScan-DS-EX Pro(H)	Shining 3D Tech Co., Ltd.	2 cameras × 5 megapixels	8 µm	Blue light	Yes
Scanner 3—Optical 3D Scanner Vinyl	Smart optics Sensortechnik GmbH (Bochum, Germany)	1 camera × 3.2 megapixels	4 µm	Blue light	Yes

**Table 3:** Dimensional accuracy assessment using AutoScan-DS-EX: root mean square (RMS) deviations and percentage of values within the acceptable deviation interval.

Scanner	Model	RMS (µm)			Acceptable Deviation (%)		
		n	Mean	Standard Deviation	n	Mean	Standard Deviation
AutoScan-DS-EX	Reference	10	0.0 ^a^	0.0	10	100.00 ^a^	0.00
	Model 1	10	120.3 ^be^	12.3	10	37.49 ^b^	2.94
	Model 2	10	122.4 ^b^	0.7	10	32.57 ^c^	0.29
	Model 3	10	119.7 ^de^	0.8	10	31.93 ^d^	0.49
	Model 4	10	127.0 ^fe^	0.0	10	31.26 ^e^	0.88
	Model 5	10	126.8 ^fe^	1.0	10	30.38 ^f^	1.01
	Model 6	10	117.2 ^ge^	0.8	10	34.38 ^g^	0.61
	Model 7	10	130.4 ^he^	0.5	10	30.84 ^ef^	0.41
	Model 8	10	139.7 ^c^	0.9	10	28.31 ^h^	0.76
	Model 9	10	129.0 ^ie^	0.7	10	30.13 ^f^	0.63
	Model 10	10	122.6 ^b^	0.7	10	34.83 ^g^	0.46

Identical letters in the vertical columns indicate no statistically significant difference, whereas different letters denote the presence of a significant difference (*p* < 0.05).

**Table 4:** Dimensional accuracy assessment using AutoScan-DS-EX Pro(H): RMS deviations and percentage of values within the acceptable deviation interval.

Scanner	Model	RMS (µm)			Acceptable Deviation (%)		
		n	Mean	Standard Deviation	n	Mean	Standard Deviation
AutoScan-DS-EX Pro(H)	Reference	10	0.0 ^a^	0.0	10	100.00 ^a^	0.00
	Model 1	10	109.2 ^b^	0.4	10	32.59 ^b^	0.33
	Model 2	10	114.6 ^c^	0.5	10	33.37 ^c^	0.51
	Model 3	10	120.0 ^d^	0.0	10	22.62 ^d^	0.16
	Model 4	10	127.8 ^e^	0.8	10	28.32 ^e^	0.74
	Model 5	10	112.0 ^f^	0.0	10	31.82 ^f^	0.25
	Model 6	10	113.0 ᵍ	0.0	10	30.80 ^g^	0.12
	Model 7	10	118.0 ^h^	0.0	10	29.27 ^h^	0.42
	Model 8	10	135.0 ^i^	0.0	10	26.74 ^i^	0.11
	Model 9	10	123.1 ^j^	0.3	10	28.33 ^e^	0.17
	Model 10	10	117.0 ^k^	0.0	10	29.60 ^h^	0.45

Identical letters in the vertical columns indicate no statistically significant difference, whereas different letters denote the presence of a significant difference (*p* < 0.05).

**Table 5:** Dimensional accuracy assessment using Optical 3D Scanner Vinyl: RMS deviations and percentage of values within the acceptable deviation interval.

Scanner	Model	RMS (µm)			Acceptable Deviation (%)		
		n	Mean	Standard Deviation	n	Mean	Standard Deviation
Optical 3D Scanner Vinyl	Reference	10	0.0 ^a^	0.0	10	100.00 ^a^	0.00
	Model 1	10	121.9 ^b^	0.7	10	33.16 ^b^	0.78
	Model 2	10	121.5 ^b^	1.9	10	31.30 ^c^	0.58
	Model 3	10	118.1 ^c^	1.9	10	32.12 ^d^	0.73
	Model 4	10	126.9 ^d^	0.7	10	29.14 ^e^	0.31
	Model 5	10	127.0 ^dfi^	2.0	9	29.29 ^e^	0.53
	Model 6	10	117.9 ^c^	0.6	10	33.21 ^di^	0.44
	Model 7	10	125.8 ^ef^	1.2	10	31.20 ^c^	0.46
	Model 8	10	138.2 ^g^	0.6	10	27.89 ^f^	0.45
	Model 9	10	128.2 ^hi^	0.8	10	31.66 ^g^	0.17
	Model 10	10	124.8 ^e^	1.5	10	33.83 ^hi^	0.73

Identical letters in the vertical columns indicate no statistically significant difference, whereas different letters denote the presence of a significant difference (*p* < 0.05).

**Table 6:** Comparison of the mean RMS values and values within the acceptable deviation interval by model, based on averaged data from the three scanners.

Model	Average RMS (µm)	Standard Deviation (µm)	Average Match (%)	Standard Deviation (%)
Sample 1	117.1	6.92	34.41	2.67
Sample 2	119.5	4.27	32.41	1.04
Sample 3	119.3	1.02	28.88	5.42
Sample 4	127.2	0.49	29.57	1.52
Sample 5	121.9	8.60	30.49	1.26
Sample 6	116.0	2.65	32.79	1.83
Sample 7	124.7	6.27	30.43	1.03
Sample 8	137.6	2.40	27.64	0.81
Sample 9	126.8	3.20	30.04	1.67
Sample 10	121.5	4.02	32.74	2.77

**Table 7:** Comparison of the mean RMS values and mean values within acceptable deviation interval by scanner, based on averaged data from the ten models.

Scanner	Mean	Std Dev
AutoScan-DS-EX, RMS (µm)	125.5	6.5
AutoScan-DS-EX, acceptable deviation in %	32.21	2.69
AutoScan-DS-EX Pro(H), RMS (µm)	118.9	7.8
AutoScan-DS-EX Pro(H), acceptable deviation in %	29.34	3.15
Optical 3D Scanner Vinyl, RMS (µm)	125.0	5.8
Optical 3D Scanner Vinyl, acceptable deviation in %	31.27	1.96

## Data Availability

The raw data supporting the conclusions of this article will be made available by the authors on request.
